# Feminizing Hormone Therapy Prescription Patterns and Cardiovascular Risk Factors in Aging Transgender Individuals in Australia

**DOI:** 10.3389/fendo.2021.667403

**Published:** 2021-07-13

**Authors:** Matthew I. Balcerek, Brendan J. Nolan, Adam Brownhill, Peggy Wong, Peter Locke, Jeffrey D. Zajac, Ada S. Cheung

**Affiliations:** ^1^ Department of Endocrinology and Diabetes, Royal Brisbane and Women’s Hospital, Brisbane, QLD, Australia; ^2^ Department of Medicine (Austin Health), University of Melbourne, Heidelberg, VIC, Australia; ^3^ Department of Endocrinology, Austin Health, Heidelberg, VIC, Australia; ^4^ Equinox Gender Diverse Clinic, Thorne Harbour Health, Fitzroy, VIC, Australia

**Keywords:** transgender, aging, estrogen, estradiol, cardiovascular

## Abstract

**Context:**

The safety and efficacy of feminizing hormone therapy in aging transgender (trans) individuals is unclear. Current recommendations suggest transdermal estradiol beyond the age of 45 years, especially if cardiometabolic risk factors are present.

**Objective:**

To evaluate feminizing hormone therapy regimens and cardiovascular risk factors in aging trans individuals.

**Design:**

Retrospective cross-sectional analysis.

**Setting:**

Primary care and endocrine specialist clinic in Melbourne, Australia.

**Participants:**

Trans individuals on feminizing therapy for ≥6 months.

**Main Outcomes Measures:**

Feminizing hormone regimens and serum estradiol concentrations by age group: (a) ≥45 years, (b) <45 years, and prevalence of cardiometabolic risk factors in individuals ≥45 years.

**Results:**

296 individuals were stratified by age group: ≥45 years (*n*=55) and <45 years (*n*=241). There was no difference in median estradiol concentration between groups (328 nmol/L *vs*. 300 nmol/L, p=0.22). However, there was a higher proportion of individuals ≥45 years treated with transdermal estradiol (31% vs. 8%, p<0.00001). Of those treated with oral estradiol, the median dose was lower in the ≥45 years group (4mg vs. 6mg, p=0.01). The most prevalent cardiometabolic risk factor in the ≥45 years group was hypertension (29%), followed by current smoking (24%), obesity (20%), dyslipidaemia (16%) and diabetes (9%).

**Conclusions:**

A greater proportion of trans individuals ≥45 years of age were treated with transdermal estradiol. Of those who received oral estradiol, the median dose was lower. This is important given the high prevalence of cardiometabolic risk factors in this age group, however cardiovascular risk management guidelines in this demographic are lacking.

## Introduction

The intersection of aging and healthcare in transgender (trans) individuals is a novel area of research, and there is a paucity of data regarding the safety and efficacy of feminizing hormone therapy in older trans individuals. Important considerations in this demographic include estradiol dose, formulation, route of administration, target sex steroid concentrations and duration of treatment.

Of particular interest in trans individuals undergoing feminizing hormone therapy, is the reversal of the traditional sex difference in the prevalence of cardiovascular disease. In the cisgender population, the prevalence of cardiovascular disease is higher in men until the age of 75 years (1). However, in the trans population, individuals on feminizing therapy (presumed male at birth) have a greater incidence of cardiovascular mortality than those on masculinizing therapy (123 per 100,000 person years vs. 15 per 100,000 person years respectively) (1). The mechanisms underlying this discrepancy are poorly understood.

Retrospective studies suggest the risk of thrombosis in trans individuals undergoing feminizing hormone therapy is highest with ethinyl estradiol, which is no longer recommended (2). There is limited information regarding the thrombotic risk of different feminizing hormone regimens, and safety data for transdermal estradiol has therefore been extrapolated from the menopausal hormone therapy literature, but notably higher doses of transdermal estradiol are used for gender affirmation (3, 4). In fact, a preliminary study suggested no apparent difference in global coagulation assays in trans people using oral or transdermal estradiol formulations (5). Nonetheless, despite the limited data, current European Network for the Investigation of Gender Incongruence (ENIGI) guidelines recommend transdermal estradiol beyond the age of 45 years (6), with continuation of the lowest dose possible to maintain adequate feminization (or in line with individual treatment goals). This is particularly prudent in aging trans individuals with established cardiovascular disease, cardiovascular risk factors and/or a history of thromboembolism.

In this retrospective analysis of individuals on feminizing hormone therapy for ≥6 months, we aimed to evaluate prescribing patterns in older (age ≥45) versus younger (age <45) trans individuals, in the context of cardiovascular risk factors that emerge with aging. We hypothesized that there would be a reduction in estradiol dose, a shift from oral to transdermal route of administration, and reduced serum estradiol concentration with aging.

## Methods

A retrospective audit of consultations for gender dysphoria was performed across two gender clinics in Melbourne, Victoria, Australia. Firstly, Equinox Gender Diverse Clinic (an Adult Primary Care General Practice), and secondly, an Adult Endocrinology Specialist Clinic. Data were collected from consecutive new consultations between 1^st^ January 2011 and 30^th^ April 2020. The audit was approved by the Austin Health Human Research Ethics Committee (LNR/17/Austin/102) and Thorne Harbour Health (THH/CREP 19/015), and informed consent was not required given the retrospective nature of the study.

This cross-sectional analysis included trans individuals (including those with a binary and/or non-binary gender identity) on feminizing hormone therapy for ≥6 months, with sufficient documentation of estradiol dose, formulation, route and serum estradiol concentration at their most recent consultation.

The primary outcome of interest was feminizing hormone regimen and serum estradiol concentration by age group: (a) ≥45 years, (b) <45 years. We chose an arbitrary cut-off of 45 years given this is the age at which ENIGI recommend changing to transdermal estradiol to minimize venous thromboembolic risk (6). Cardiometabolic risk factors were collected in the ≥45 years group (hypertension, dyslipidaemia, diabetes, obesity and smoking history), as well as history of ischaemic heart disease, cerebrovascular disease, or venous thromboembolic disease.

Estradiol concentration was measured using immunoassays available as standard care for clinical decision-making and only laboratories accredited by the National Association of Testing Authorities (NATA) were used.

Statistical analyses were performed using STATA version 16.1 software (StataCorp. 2019. *Stata Statistical Software: Release 16*. College Station, TX: StataCorp LP). As data were not normally distributed, median (IQR) are presented. Differences between groups were tested using the Mann-Whitney U test or Kruskall-Wallis test. Fisher’s exact test was used for the proportion of individuals treated with oral vs. transdermal estradiol by age group. A *p*-value of <0.05 was considered statistically significant.

## Results

Data were collected from 390 individuals of whom 296 had been treated with estradiol for at least 6 months and had adequate data available for analysis. Of these, 55 individuals were aged ≥45 years. Individuals treated with ethinyl estradiol (*n*=12) or anti-androgen monotherapy (*n*=3) were excluded from this analysis.

Median age of individuals was 26.3 (22.1, 38.6) years and duration of feminizing therapy 25.2 (10.7, 32.9) months. In all, 254 (85.8%) individuals were treated with oral estradiol. Median estradiol concentration achieved was 325 (233, 428) pmol/L.

### Age ≥45 Years

**Figure 1:**
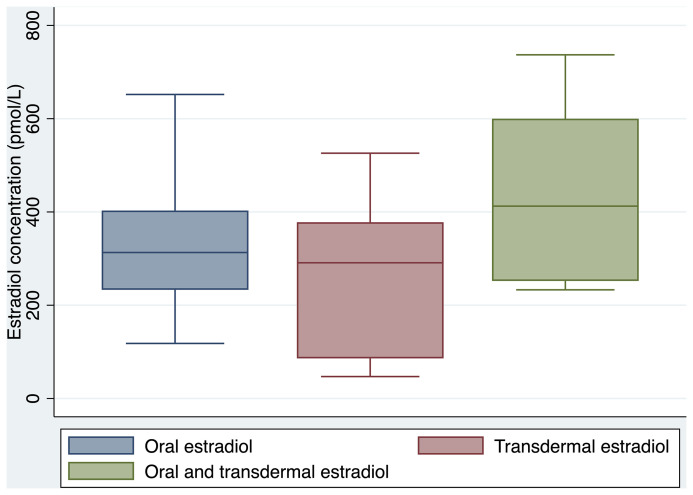
Serum estradiol concentration by estradiol formulation (age ≥45 years).

Clinical and laboratory parameters are outlined in **Table 1**. Oral estradiol valerate was the most frequently prescribed formulation (58%), followed by transdermal estradiol (31%), and combination oral and transdermal estradiol (11%). There was no significant difference between groups in serum estradiol concentration achieved (322 pmol/L for oral estradiol, 293 pmol/L for transdermal estradiol, and 413 pmol/L for combination oral and transdermal estradiol, overall p=0.108). Box plots of serum estradiol concentration by estradiol formulation are shown in **Figure 1**.

**Table 1:** Clinical Characteristics (≥45 years age group).

	Overall(n = 55)	Oral estradiol(n = 32)	Transdermal estradiol(n = 17)	Combination therapy (oral and transdermal)(n = 6)	P-value^#^
Age (years)	52.8 (49.7, 58.3)	52.5 (47.6, 55.6)	59.1 (52.5, 63.1)	48.1 (46.0, 52.0)	0.014
BMI (kg/m^2^)	27.1 (25.0, 30.1)	27.7 (25.5, 29.4)	29.6 (24.8, 34.1)	25 (24.3, 27)	0.358
Duration of feminizing therapy (months)	69.0 (45.9, 134.9)	78.3 (52.2, 168.9)	48.0 (23.8, 87.7)	132.6 (49.7, 240.5)	0.076
Estradiol dose	–	4 (4-6) mg	Patch 62.5 (50-100) mcg Gel 1 (1-1.25) mg	Oral 6 (2.5-8) mg Patch 50 (50-100) mcg Gel 1 (1-1) mg	–
Estradiol concentration (pmol/L)	300 (222, 414)	322 (233, 414)	292.5 (133.3, 361)	412.5 (281.8, 563.5)	0.108
SHBG (nmol/L)	77 (48, 107)	88 (71, 126)	46 (38, 63)	93 (62, 106)	0.005

Median (IQR) are shown. BMI, body mass index; SHBG, sex hormone binding globulin. ^#^P-value from Kruskal-Wallis test.

There was no significant difference in median serum estradiol concentration when comparing individuals with or without previous orchidectomy and/or genitoplasty (264 nmol/L *vs*. 316 nmol/L respectively, p=0.55). Individuals with previous orchidectomy and/or genitoplasty were treated with lower oral estradiol doses (3.4 mg *vs*. 5.7 mg, p=0.003).

Trans individuals prescribed transdermal estradiol were older than those prescribed oral estradiol (59.1 years *vs*. 52.5 years respectively, p=0.03). A strong association was observed between sex hormone binding globulin (SHBG) concentration and route of estradiol administration, with a median SHBG concentration of 46 nmol/L in the transdermal estradiol group, compared to 88 nmol/L in the oral estradiol group (p=0.0012).

Cyproterone acetate (CPA) was the most commonly prescribed anti-androgen (*n*=15), followed by spironolactone (*n*=11) and goserelin (*n*=1), with a median serum testosterone concentration of 0.5 (0-4) pmol/L. Median dose of CPA and spironolactone were 25 (18.75-25) mg and 100 (50-150) mg respectively, and there was no significant difference in median serum testosterone concentration achieved (0.4 vs. 0.5 nmol/L respectively, p=0.09). Eight individuals (15%) were co-prescribed a progestogen; two individuals were treated with micronised progesterone, and six with medroxyprogesterone acetate (MPA).

The median body mass index (BMI) of all individuals was in the overweight range at 27.1 (25.0, 30.1) kg/m^2^, whereas individuals treated with transdermal estradiol had median BMI of 29.6 (24.8-34.1) kg/m^2^. Cardiometabolic risk factors are shown in **Table 2**. The most prevalent cardiovascular risk factor was hypertension (29%), followed by current smoking (24%), obesity (20%), dyslipidaemia (16%) and diabetes (9%). Of note, there was a higher proportion of individuals treated with transdermal estradiol with a history of dyslipidaemia (p=0.0004).

**Table 2:** Cardiometabolic risk factors (≥45 years age group).

	Overall (n=55)	Oral estradiol (n=32)	Transdermal estradiol (n=17)	Combination therapy (oral and transdermal) (n=6)	P-value^#^
Current smoker	13 (24%)	7 (22%)	4 (24%)	2 (33%)	0.810
Ex-smoker	12 (22%)	8 (25%)	2 (12%)	2 (33%)	0.455
Dyslipidaemia	9 (16%)	1 (3%)	8 (47%)	0 (0%)	0.0004
Diabetes	5 (9%)	2 (6%)	3 (18%)	0 (0%)	0.368
Hypertension	16 (29%)	8 (25%)	6 (35%)	2 (33%)	0.684
Obesity (BMI > 30kg/m^2^)	11 (20%)	6 (19%)	4 (24%)	1 (17%)	0.885
IHD	3 (5%)	1 (3%)	2 (12%)	0 (0%)	0.489
CVA	2 (4%)	1 (2%)	1 (6%)	0 (0%)	0.999
VTE	1 (2%)	0 (0%)	1 (6%)	0 (0%)	0.418

^#^P-value from Fisher’s exact test for categorical variables. BMI, body mass index; IHD, ischaemic heart disease; CVA, cerebrovascular disease; VTE, venous thromboembolic disease

### Comparison to Group Aged <45 Years

**Figure 2:**
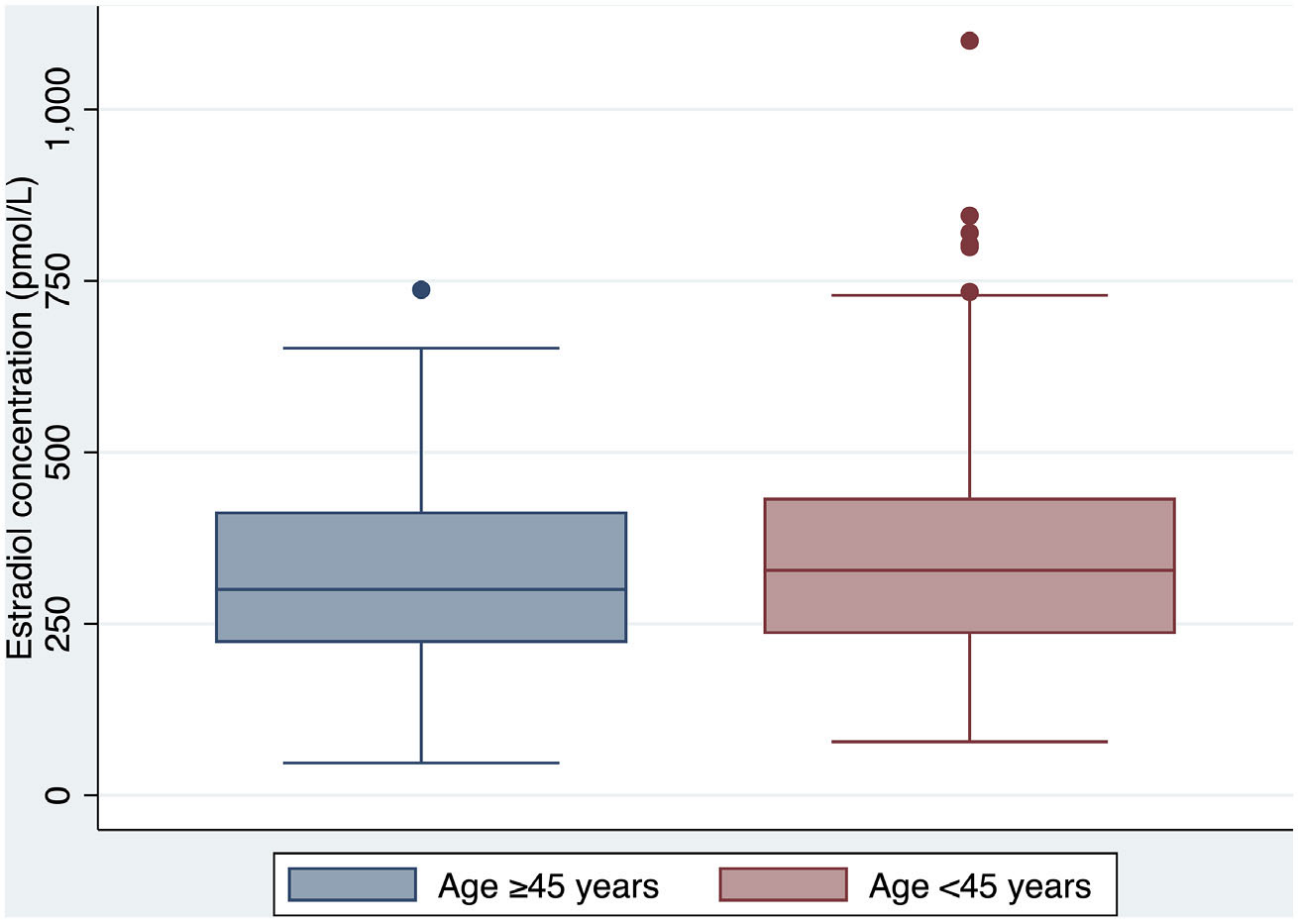
Serum estradiol concentration by age group (≥45 *vs* <45 years).

Clinical and laboratory parameters of the <45 years group are outlined in **Table 3**. There was no difference in serum estradiol concentration achieved between the ≥45 and <45 years age groups (300 nmol/L *vs*. 328 nmol/L respectively, p=0.22) (**Figure 2**). However, there was a significant difference in the proportion of individuals treated with transdermal estradiol between the two age groups (31% in the ≥45 years group *vs*. 8% in the <45 years group, p<0.00001).

**Table 3:** Clinical Characteristics (<45 years age group).

Age (years)	24.6 (21.5, 28.9)
BMI (kg/m^2^)	24.2 (21.6, 28.5)
Duration of feminizing therapy (months)	23.8 (15.2, 32.4)
Estradiol dose	Oral 6 (4-8) mg
Estradiol concentration (pmol/L)	328 (237, 434)

Median (IQR) are shown. BMI, body mass index.

The median oral estradiol dose was 4 (4-6) mg in the ≥45 years group, which was lower than the median dose of 6 (4-8) mg in the <45 years group (p=0.01). Individuals in the <45 years group were more commonly prescribed a progestogen (32% *vs*. 15%).

## Discussion

In this retrospective cross-sectional analysis of feminizing hormone therapy regimens for trans individuals attending gender clinics in Australia, there was a higher proportion of individuals over the age of 45 years who were treated with transdermal estradiol compared to those aged less than 45 years (31% *vs*. 8%, p<0.00001). This is in keeping with the ENIGI Guidelines, which recommend transdermal estradiol in patients older than 45 years (6). There remain a considerable 58% treated with oral estradiol. Of those treated with oral estradiol, the median dose was lower in the **≥**45 years group (4mg vs. 6mg, p=0.01), and the SHBG was higher (88nmol/L *vs*. 46nmol/L, p=0.004).

### Comparison to Previous Studies

To our knowledge, there is no published data specifically evaluating feminizing hormone regimens and estradiol concentrations in older trans individuals. Previous studies that have enrolled older trans women have noted increased mortality from ischaemic heart disease in those aged 40-64 years (2), however they have not reported on estradiol dose, formulation, route and concentration with aging. Furthermore, current recommendations for aging trans women are predominantly expert opinion pieces (7), with many recommendations being extrapolated from the menopausal hormone therapy literature. Our study provides an insight into the prescribing patterns of experienced Australian transgender healthcare providers, in the context of cardiovascular risk factors that emerge with aging.

### Current Guidelines for Feminizing Hormone Therapy in Aging Transgender Individuals

There is no evidence to guide continuation or cessation of feminizing hormone therapy in aging trans individuals (8). Recommendations are mainly speculative and reflect experience with hormone replacement in postmenopausal women. ENIGI guidelines recommend changing to transdermal preparations beyond the age of 45 years due to the ‘first pass effect’ of the liver and associated thrombotic risk of oral estrogens. Other expert groups suggest the lowest effective dose, with preference for transdermal formulation in those beyond 50-55 years, due to potential cardiovascular side effects becoming more apparent (7). Whilst ranges of estradiol are provided by some guidelines, these are based upon expert opinion and their applicability to older individuals is unclear (9, 10).

Complete discontinuation of hormones in aging trans individuals, especially in those who have undergone orchidectomy, may lead to profound bone loss (10). Additionally, in people who have not undergone orchidectomy, withdrawal of feminizing hormone therapy may result in virilization (8). As such, continuation of estradiol in an ‘age-appropriate’ dose beyond the age of 50 years is recommended (11).

In terms of our study population, 58% of patients over the age of 45 years were prescribed oral estradiol, with an additional 11% being prescribed combination oral and transdermal therapy estradiol. 31% were prescribed transdermal monotherapy. Whilst there was a significant transition from oral to transdermal therapy beyond the age of 45 years, preference for the oral route as first-line therapy may reflect patient/clinician preference or practical difficulties with transdermal patches, such as adherence issues due to excessive hair or sweat on the skin (12).

Overall, the risks and benefits of continuing versus withdrawing feminizing hormone therapy is an individualised discussion, and shared decision making is recommended (8). Unless there are clear contraindications, feminizing therapy should not be withheld or withdrawn from trans individuals based purely on age (7).

### Extrapolations From the Menopausal Hormone Therapy Literature

No prospective, randomized controlled trials have been performed comparing oral versus transdermal estradiol in postmenopausal women, nor the trans population (12). A previous large population-based study of postmenopausal women demonstrated a similar increased risk of stroke when comparing oral estradiol (both high and low dose) to high-dose transdermal estradiol (>50mcg) (13). However, this risk has not been borne out in subsequent meta-analyses, which have suggested transdermal estrogens carry minimal or no thrombotic risk, even in women with a prior history of thrombosis (3, 4). Transdermal estrogens have negligible effects on haemostatic variables, which has led many authors to recommend this route in postmenopausal women with thrombotic risk factors (14).

This concept has been extrapolated to the trans population, however, notably trans people using feminizing hormones typically use far higher doses than cisgender women using menopausal hormone therapy (15). In fact, a study of 26 trans people on estradiol therapy demonstrated hypercoagulable global coagulation assay parameters compared with cisgender men with a shift towards cisgender female parameters (5). There was no difference between transdermal or oral routes of administration with both groups showing hypercoagulable global coagulation assay parameters. As such, the benefit of using transdermal estradiol in older trans women to minimise thrombosis risk factors is unclear. As a harm minimisation approach, as there is a potential dose-response relationship (12), the lowest transdermal dose that maintains feminization should be used.

The effect of estrone, the main metabolite of oral estradiol, on thrombin generation has also been posited as a potential mediator of VTE risk with oral estradiol therapy in postmenopausal women (16). Higher estrone concentrations have been demonstrated in trans women and non-binary adults taking sublingual estradiol compared to transdermal or injectable preparations (17). However, the role of monitoring serum estrone concentrations in individuals taking feminizing hormone therapy is yet to be determined and requires further investigation.

An expected finding is the higher SHBG concentration in those treated with oral estradiol (18). SHBG is elevated in the presence of estrogen, more so with oral estradiol than parenteral routes, due to first pass hepatic metabolism (19). SHBG has been postulated as a marker of estrogenicity and a potential surrogate indicator of VTE risk (19). The low prevalence of VTE in our study precluded analysis of this biochemical marker in relation to VTE risk, however future studies are warranted to further evaluate SHBG concentration in trans patients receiving feminizing hormone therapy (20).

### Prevalence of Cardiovascular Disease in Transgender Individuals Receiving Feminizing Hormone Therapy

The relationship between feminizing hormone therapy and cardiovascular risk in trans women is complex and is influenced by many factors including estradiol formulation, route of administration, dose, and baseline cardiovascular status (21). In the general population, cisgender men have a cardiovascular survival disadvantage relative to cisgender women up until the age of 65-75 years (7). However, in the trans population, individuals on feminizing therapy have a greater incidence of cardiovascular mortality than individuals on masculinizing therapy. This survival disadvantage was demonstrated in a recent large cohort study (22), which highlighted an increased incidence of VTE and ischaemic stroke in trans women compared to both cisgender men and women.

Increased mortality from ischaemic heart disease has also been demonstrated in trans individuals aged 40-64 years receiving feminizing therapy for a median duration of 18.5 years (2). It should be noted that most cardiac events occurred in individuals treated with ethinyl estradiol (2), which is no longer recommended in consensus guidelines (10).

The risk of oral estradiol versus transdermal estradiol is less clear, as there have been no prospective studies comparing these two formulations. A previous cross-sectional study demonstrated an increased rate of myocardial infarction in trans women after a median duration of 6 years of feminizing therapy [prescribed estradiol formulations were transdermal estradiol (49.1%), estradiol valerate (42.5%) and ethinyl estradiol (3.2%)] compared to their cisgender counterparts (21). The mean age at time of myocardial infarction was 48 years, and the majority of trans women with cardiovascular events had one or more cardiovascular risk factors, mainly smoking.

A number of changes in surrogate cardiovascular risk markers occur after commencing feminizing hormone therapy, including increased weight, visceral fat, total body fat, reduced insulin sensitivity, and potentially increased systolic and diastolic blood pressure (7, 23). Endocrine Society guidelines suggest cardiovascular risk factors be treated as they emerge, in accordance with established guidelines (10), and other groups advocate managing hypertension, hypercholesterolaemia, diabetes and smoking before initiating feminizing therapy as a risk mitigation strategy (9, 21).

The underpinnings of the reversed sex difference in cardiovascular mortality are yet to be fully elucidated, particularly what degree of risk is genetically determined, and whether this risk is modified by changes in the sex steroid milieu (7). Further research is required to investigate possible mechanisms contributing to the increased prevalence of cardiovascular disease in trans women receiving feminizing hormone therapy.

### Cardiovascular Risk Factors in Transgender Individuals Receiving Feminizing Hormone Therapy Compared to the General Australian Population

The most prevalent cardiovascular risk factor in our study was hypertension, followed by current smoking. Nearly 1 in 4 patients in the ≥45 years group were current smokers (24%). This is considerably higher than recent Australian population data highlighting a smoking prevalence of 16.9% in the general population in persons aged 45-54 years (males 19.3%, females 14.7%) (24). Smoking rates in trans individuals aged ≥45 in our study were also higher than a recent cross-sectional survey of Australian trans adults (current smoking 15%), noting that this survey reflected a younger demographic with a median age of 28 years (25).

Obesity was the third most prevalent cardiovascular risk factor in our study population. The median BMI in the ≥45 years group was in the overweight range 27.1 (25.0-30.1) kg/m^2^. This is considerably higher than the median BMI of 24.4 kg/m^2^ in a previous Belgian cross-sectional study evaluating 214 trans women with a median age of 43.7 years (21). However, the median BMI of the ≥45 years group was not considerably higher than the median BMI of Australians aged 45-54 years based on 2017-2018 Australian Institute of Health and Welfare data (26).

Almost half of the trans women in the transdermal estradiol group (47%) had dyslipidaemia, compared to just 3% of patients in the oral estradiol group (p=0.0004). A recent systematic review highlighted the effect of oral estrogens on increasing triglycerides (27), which may be one factor influencing the preference for prescribing transdermal therapy to older trans women with dyslipidaemia in our study.

Irrespective of the low prevalence of cardiovascular events in our study, the disproportionately high prevalence of cardiovascular risk factors highlights an at-risk population, and we therefore advocate for proactive and aggressive management of modifiable risk factors to mitigate the risk of developing overt cardiovascular complications.

### Limitations

There are inherent limitations to this study given the retrospective cross-sectional design. These include missing data (duration of feminizing therapy 13/55, estradiol concentration 5/55, SHBG 7/55), lack of randomisation of estradiol formulation, and reliance on clinical records to determine prevalence of cardiovascular risk factors. Similarly, data regarding cardiovascular risk factors and cardiovascular disease were not collected in the <45 years age group. Potential confounders include prescriber preference for estradiol formulation, individual adherence with prescribed feminizing therapy, as well as adherence with any other prescribed therapy for cardiovascular risk reduction. We were unable to determine prescriber or patient reasons for choice of therapy.

We examined prescribing patterns across two clinics experienced in trans health care, an Adult Primary Care General Practice and an Adult Endocrinology Specialist Clinic, both located in Melbourne, Australia. Prescribing patterns were in accordance with National Guidelines (9), and are likely to reflect overall trends in Australia. It should be noted that oral and transdermal estradiol are available on the Pharmaceutical Benefits Scheme (PBS) in Australia, so prescribing patterns may vary between countries.

Estradiol concentrations reported represent a single point in time, acknowledging that there can be significant intraindividual variability between samples. Estradiol concentrations were also measured *via* different immunoassays as standard care. Furthermore, we did not have data describing feminine physical characteristics achieved by individuals.

There were small patient numbers treated with combination oral and transdermal estradiol therapy, however this is representative of feminizing hormone prescribing in Australia (12). Similarly, there were a limited number of individuals treated with estradiol for greater than 5 years, and the relationship between duration of therapy and prescribing patterns could be a consideration for future research. The prevalence of VTE and cardiovascular events were low, which can lead to imprecision of estimates, however this is one of the largest cohorts to date specifically evaluating feminizing hormone regimens and cardiometabolic risk factors in aging trans women.

## Conclusions

A greater proportion of trans women ≥45 years of age are treated with transdermal estradiol, and of those who were not treated with transdermal therapy, the median oral estradiol dose was lower. This highlights a preference for transdermal therapy in aging trans women, however this is not universal given the lack of evidence comparing the safety of different routes of administration on thrombosis and cardiovascular risk. Well-designed prospective studies with larger subject numbers and longer duration of follow up are required to evaluate the safety and efficacy of modern feminizing hormone therapy regimens in trans individuals across their lifespan.

## Data Availability Statement

The raw data supporting the conclusions of this article will be made available by the authors, without undue reservation.

## Ethics Statement

The studies involving human participants were reviewed and approved by Austin Health Human Research Ethics Committee. Written informed consent for participation was not required for this study in accordance with the national legislation and the institutional requirements.

## Author Contributions

MB and BN contributed to conception, design, statistical analysis and drafting of the manuscript, which was overseen by AC. All authors contributed to the article and approved the submitted version.

## Funding

BN is a recipient of a National Health and Medical Research Council Postgraduate Scholarship (#2003939). AC is supported by an Australian Government National Health and Medical Research Council Early Career Fellowship (#1143333) and receives research support from the Viertel Charitable Foundation Clinical Investigator Award, Endocrine Society of Australia, Austin Medical Research Foundation, and the Royal Australasian College of Physicians Foundation.

## Conflict of Interest

The authors declare that the research was conducted in the absence of any commercial or financial relationships that could be construed as a potential conflict of interest.

## Abbreviations

BMI, body mass index; CPA, cyproterone acetate; MPA, medroxyprogesterone acetate; SHBG, sex hormone binding globulin; trans, transgender; WHI, Women’s Health Initiative.
